# Implementing the analogous neural network using chaotic strange attractors

**DOI:** 10.1038/s44172-024-00242-z

**Published:** 2024-07-15

**Authors:** Bahadır Utku Kesgin, Uğur Teğin

**Affiliations:** Department of Electrical and Electronics Engineering, Koç University, Istanbul, 34450 Türkiye

**Keywords:** Statistical physics, thermodynamics and nonlinear dynamics, Computer science, Electrical and electronic engineering

## Abstract

Machine learning studies need colossal power to process massive datasets and train neural networks to reach high accuracies, which have become gradually unsustainable. Limited by the von Neumann bottleneck, current computing architectures and methods fuel this high power consumption. Here, we present an analog computing method that harnesses chaotic nonlinear attractors to perform machine learning tasks with low power consumption. Inspired by neuromorphic computing, our model is a programmable, versatile, and generalized platform for machine learning tasks. Our mode provides exceptional performance in clustering by utilizing chaotic attractors’ nonlinear mapping and sensitivity to initial conditions. When deployed as a simple analog device, it only requires milliwatt-scale power levels while being on par with current machine learning techniques. We demonstrate low errors and high accuracies with our model for regression and classification-based learning tasks.

## Abstract (web summary)

Bahadır Utku Kesgin and Uğur Teğin propose using a Lorenz attractor as a nonlinear transfer function for neural network nodes. They design a power-efficient electrical circuit and use them for regression and classification test tasks.

## Introduction

Current computing methods and hardware limit machine learning studies and applications regarding speed, data resolution and deployed platforms. Particularly, the power consumption of artificial neural networks started to raise questions regarding its impact on the environment. Recent studies indicate that the carbon emissions of training a complex transformer learning model are roughly equivalent to the lifetime carbon emissions of five cars^1^, and training a famous language model consumed the energy required to charge 13,000 electric cars fully^2^. Several computing paradigms are proposed for machine learning studies to decrease training times and, therefore, the energy consumption issue. Among them, reservoir computing^3,4^ offers a promising path by using nonlinear systems with fixed weights to process information in high dimensional space. Various neuromorphic devices^5^ were proposed to surpass chronic performance issues of conventional computing and high-power consumption issues. Optical computing methods^6–8^ and electronic memristive devices^9–11^ were introduced as powerful reservoir computing platforms. The concept of fixed nonlinear high-dimensional mapping is of usual practice in several areas of machine learning, such as extreme learning machines^12^ and support vector machines^13,14^.

In machine learning studies, chaotic systems were mainly employed as targets to learn dynamical systems^15–21^. Chaos theory examines deterministic but unpredictable dynamical systems that are extremely sensitive to initial conditions. These systems commonly occur in nature, inspiring art, science, and engineering^22^. Also, chaotic spiking dynamics of neurons have inspired several neuromorphic machine learning applications^23,24^. In the past, chaotic systems were proposed for Boolean computation and data processing, forming the concept of chaos computing. Early chaos computing devices operated one-dimensional chaotic maps to perform logic operations^25,26^. These dynamical systems were also suggested for reservoir computing but used in a stable state just below the bifurcation point, where order transitions to chaos^27^. Operating in a stable state, such systems could not benefit from chaos in learning and information processing for machine learning purposes. Following these attempts, systems with weakly chaotic architecture were proposed^28,29^. However, these models and other similar approaches could not demonstrate competent performances^30^.

**Fig. 1:**
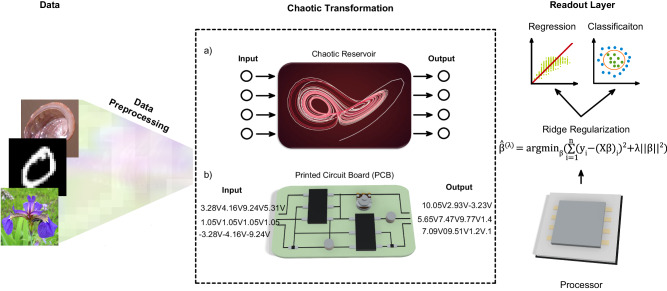
Schematic displaying the architecture of our model. Input data is preprocessed and encoded as initial voltages for the circuit performing analog computation of Lorenz attractor. **a** Displays the chaotic reservoir. **b** Displays the chaotic transformation applied by the circuit. After the chaotic transformation of data, output voltages are transferred to a processing device as reservoir output. Via the device, the last layer is performed, mainly ridge regression and classification, completing the learning process.

Here, we propose an analog computing method based on controllable chaotic learning operators to perform high-dimensional nonlinear transformations on input data for machine learning purposes. Our method benefits circuits designed to compute chaotic strange attractors for reservoir computing purposes, as demonstrated in Fig. 1. The methods section elaborately outlines the low-power computation of chaotic attractors using simple analog circuits. At present, solely chaotic attractors enable high-performance controllable analog machine learning in conjunction with milliwatt-scale power consumption. This advance originates from our method containing notable properties of chaos, thus eliminating the need for high power to process information. Since minor differences amplify and evolve in chaotic attractors, chaotic processors in our method evidently improve machine learning.

While previously reported physical reservoir computing hardware lacks flexibility, we introduce a controllable model by increasing overall versatility. Achieving this versatile platform allows us to enhance overall learning accuracy for various learning tasks through optimization. Our computing method intrinsically offers smaller footprints with power consumption levels as low as a milliwatt scale while preserving high accuracies. By providing complex and chaotic dynamics for the nonlinear transformation of data, our model performs on par with neural networks operating on conventional computing platforms. We present the generalizability of our approach by testing a wide variety of machine learning tasks, including image classification, and achieve high accuracies, reaching up to %99 for several tasks. To further show the efficacy of our approach, we also juxtapose our findings with a widely acknowledged reservoir computing technique and with memristive machine learning operators. Later, we explore how sensitivity to initial conditions in chaotic attractors improves learning accuracy and determines the power consumption required for training. Our method is a controllable, chaotic analog learning device that offers versatile and sustainable machine learning without compromising learning performance.

## Results

### Input/Output encoding and selection of the optimal attractor

As illustrated in Fig. 1, our computing approach consists of input data, chaotic reservoir, and readout layers. We first apply a chaotic transformation to input data via a chaotic circuit and then utilize a simple digital readout layer to complete the learning process. Chaotic systems are extremely sensitive to initial conditions such that their response drastically changes even in nano-scale perturbations. Due to this sensitivity, it is crucial to set an encoding method that makes the attractor transform input samples in a way that makes it easier for the classification algorithm to make distinctions between classes. It is also important to preserve the integrity of the physical model to perform stable computation. We decide to input our data as initial conditions of the attractor. As we scale our data using z-score normalization, the initial conditions we use as inputs land in a scale that does not vitiate the physical model (see Supplementary Note 4 and Supplementary Fig. 4). After the chaotic transformation is applied to our samples, we feed the transformed matrix to the regression or classification algorithm (see Methods Numerical simulations for specific algorithms).

The pattern and average separation pace between each chaotic attractor’s close points are distinctive properties. We select six different chaotic attractors to evaluate how these unique properties translate into machine learning. We employ a nonlinear regression task on a randomly generated Sinus cardinal dataset (see Methods Data preprocessing). We select the well-known Lorenz attractor^31^, Rössler attractor^32^, Burke-Shaw system^33^, Sprott attractor^34^, Chen’s system^35^, and Chua’s Circuit^36^ for this test. Our test attractors transformed randomly generated points for one hundred iterations and tried to predict Sinus Cardinal function values corresponding to the transformed sample. After recording the lowest root mean squared error (RMSE) amongst iterations, we sort each result from smallest to largest RMSE value. Lorenz attractor was the most successful attractor with a RMSE of 0.143. We decide to proceed with further tests only using the Lorenz attractor and using the iteration with the lowest error after 100 iterations (see Supplementary Table 1 and Supplementary Note 4 for details).

### Sinus cardinal regression

To assess the potential performance of machine learning with chaotic attractors, we run a simple regression task on a dataset of randomly generated samples and their values after the Sinus Cardinal function. Sinus Cardinal (Sinc), as a nonlinear function, is a commonly preferred initial test for extreme machine learning and reservoir computing. By evaluating the linear regression performance of the processed Sinc dataset, the presented models demonstrate whether the model can perform the nonlinear transformation of data. In aforesaid benchmarking tests, we measure the vanilla RMSE of Lorenz attractor as 0.143. We apply the Bayesian Optimization algorithm (see Methods Numerical simulations for details) to determine the best values for Lorenz system parameters to minimize error and improve model performance. After completing three separate optimizations, we select the values that lead to minimum error (σ = 10, β = 8/3, and $\rho$= 97). We use these coefficients in further tasks except the Abalone dataset, where we applied a separate optimization. After the optimization, an RMSE of 0.105 is achieved.

To further decrease learning error and test different configurations of our model, we add another layer that will apply the chaotic transformation to the input variable. First, two parallel Lorenz Attractor layers with different ρ values transform the same input simultaneously. These two distinct outputs are concatenated into a single matrix, and this matrix undergoes the learning process. Keeping σ and ρ as fixed variables, we apply Bayesian Optimization to determine optimal ρ values for our transformers. After the optimization process, we decrease model RMSE down to 0.03 (ρ1 = 94.087, ρ2 = 36.867). (see Supplementary Fig. 1) By using nested loops error of the model can be decreased notably due to the dimensionality expansion and impact of chaotic transformers with different chaos parameters (see Largest Lyapunov exponent and accuracy). In the remainder of our study we only test our model by deploying a single transformer per variable to accurately characterize the properties of our analog learner without adding another chaotically complex variable.

### Abalone dataset

Moving on with a relatively more complex and multivariable regression task, we test our chaotic model in the abalone dataset. This dataset, taken from ref. ^37^, is composed of the eight physical measurements of sea snails and their ages. We normalize the ages on a scale between 0 and 1. We apply z-score normalization and deploy chaotic transformation with a single transformer to every single variable. We use Bayesian optimization to find the optimal parameters of the Lorenz transformer. After optimization, we achieve remarkable accuracy (RMSE 0.072884) with parameters: σ = 10, β = 2.667, ρ = 64.917. (see Supplementary Fig. 1 for the result and Methods Numerical simulations for optimization details).

### Iris dataset

**Fig. 2:**
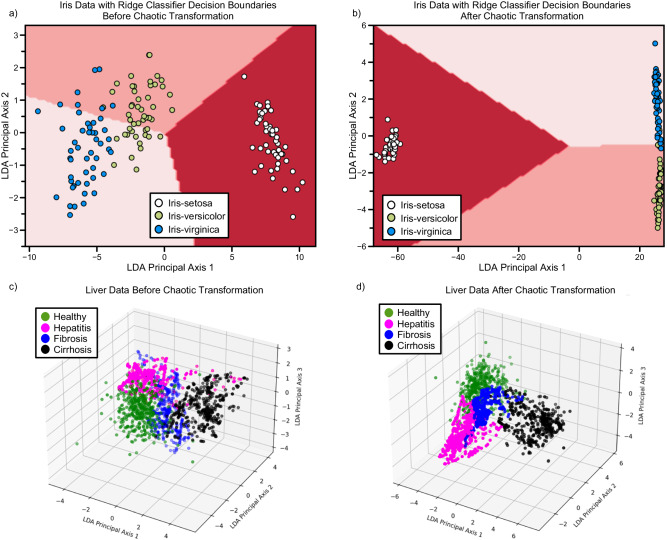
Impact of chaotic nonlinear transformation on the decision boundaries and the data points. Decision boundary of ridge classifier in Iris dataset before (**a**) and after (**b**) the chaotic transformation. Distribution of datapoints of Liver dataset before (**c**) and after (**d**) the chaotic transformation.

**Fig. 3:**
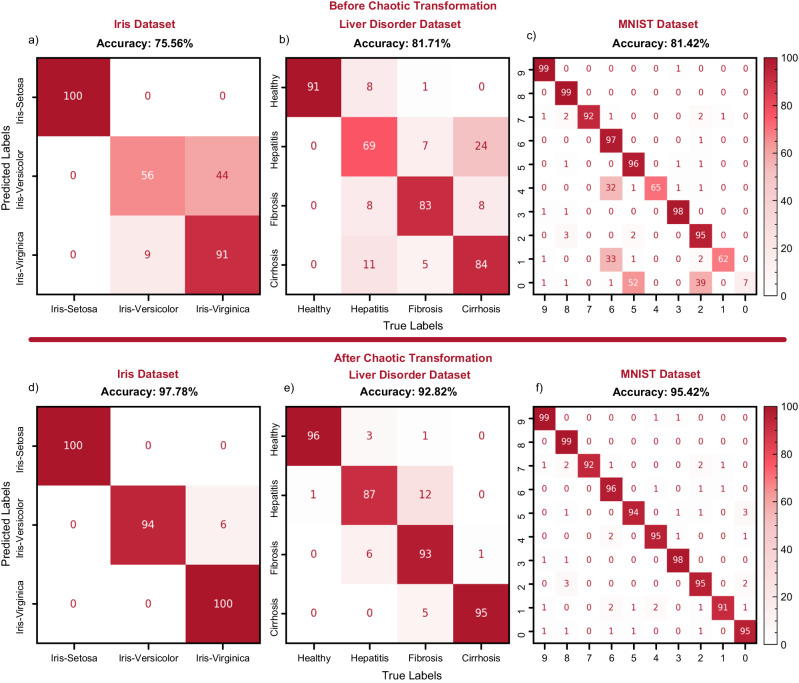
Confusion matrices of Ridge classifier accuracy in three classification tasks before and after chaotic transformation. The subfigures **a**–**c** represent accuracies before chaotic transformation was applied and the subfigures **d**–**f** represent accuracies after chaotic transformation. Confusion matrices of each dataset are represented as follows: Iris dataset (**a**, **d**), Liver Disorders Dataset (**b**, **e**), and MNIST Dataset (**c**, **f**). Confusion matrices are normalized row-wise (see Methods Data preprocessing for details).

We move on with classification tasks to challenge our model. The Iris dataset is one of the classical datasets that assess linear and nonlinear classification abilities. The dataset from ref. ^38^ consists of four physical measures of iris flowers from three distinct species. While one class, iris-setosa, is linearly separable from the other two classes, iris-versicolor and iris-virginica require nonlinear applications to be separated. Thus, with a relatively small dataset we evaluate our model both on linear and nonlinear classification. We employ Ridge classification as the last layer because it is a simple and linear method that is fast to execute. Ridge regularization is important to prevent overfitting especially when mapping data into high dimensions. Changing the usual method for visualizing classifier decision boundary, we use Linear Discriminant Analysis (LDA) to raw and transformed data (see Fig. 2). Using LDA, we retrieve 2D matrices for raw and transformed data and perform Ridge classification to these 2D matrices. A high accuracy of 97.78% is achieved, gaining about 18% over model accuracy before chaotic transformation (80.00%). After chaotic transformation, samples that belong to linearly non-separable classes (iris-versicolor and iris-virginica) all clustered almost perfectly (see Fig. 2). As a result, the linear classifier we utilize can make an almost perfect classification. We also test other classifiers for benchmarking (see Methods Numerical simulations and Supplementary Table 2 for details). A drastic increase in test accuracy of linearly inseparable classes is demonstrated in confusion matrices (see Fig. 3).

### Liver disorders dataset

For this classification task, we test our methods in the liver disorders dataset. This dataset, taken from ref. ^39^, comprises 12 features in blood samples taken from healthy people, hepatitis patients, fibrosis patients, or cirrhosis patients. After obtaining an even dataset (see Methods Data preprocessing for details), we employ the same chaotic transformation method to our features. With chaotic transformation, we report an increase in the ridge classifier accuracy by about 11% from 81.71% to 92.82% and achieve an accuracy of 98.84% with Linear SVM (see Supplementary Table 2). Although this dataset provides an extra challenge to model after the Iris dataset due to the intertwined distribution of classes with chaotic transformation, like previous results, classes are well-clustered, and decision boundary lines are easier to draw (see Fig. 2). Also, substantial improvement in the accuracies of every single class is displayed in confusion matrices (see Fig. 3). We also employ an extreme learning machine for benchmarking to this task. Extreme learning machines are the closest alternative to our model regarding the reservoir model and reduced trainable parameters. Our model surpasses extreme learning machines with the same number of learning parameters by 10% in test set accuracy (see Supplementary Fig. 6 and Supplementary Note 6 for confusion matrices and details).

### MNIST dataset

We test our model for image classification after proving strong performance in numerical datasets. MNIST dataset^40^ contains 70,000 samples (60,000 training, 10,000 testing) of 10 handwritten digit classes. For this task, 28 × 28 images are flattened without any normalization, and a fast algorithm for dimensionality reduction (see Methods Data preprocessing for details) is employed as a form of preprocessing. After reducing dimensions of each flattened images from 1 × 784 to 1 × 7, we perform classification and set a baseline accuracy. After chaotic transformation, the accuracy of this Ridge classifier increase 81.42% to 95.42%. We again utilize an extreme learning machine to this task for benchmarking. Our model surpasses extreme learning machines with the same number of learning parameters by 6.91% in test set accuracy. Our model also surpasses multi-memristive synapse neural network architecture by 6.32% in test set accuracy (see Supplementary Table 2 and Supplementary Fig. 7 for details). Such a drastic increase in accuracy highlights the effect of chaotic nonlinear transformation one more time.

### Largest Lyapunov exponent and learning accuracy

Next, we investigate the impact of sensitivity to initial conditions on our model’s performance in machine learning tasks. We set the Largest Lyapunov Exponent (λ)(LLE)^41^ to measure the pace of separation in a chaotic system. An LLE that is larger than 0 indicates a chaotic system, and a larger LLE corresponds to faster separating points.

**Fig. 4:**
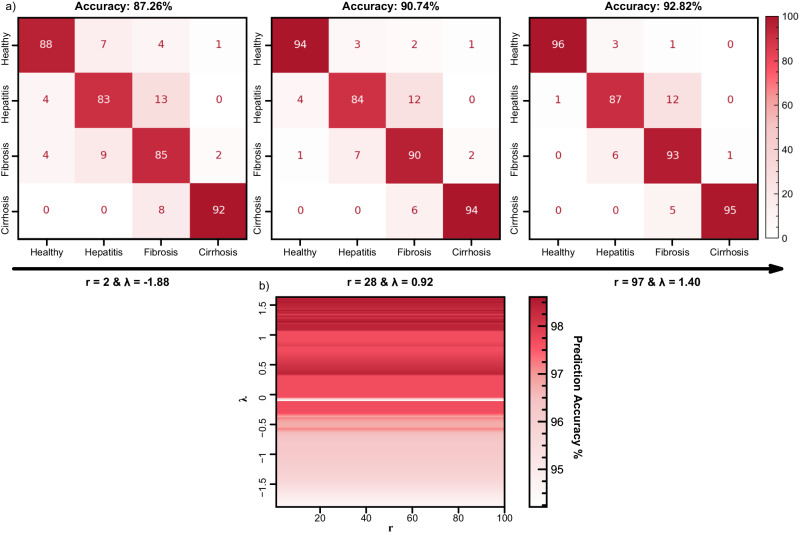
Relation between sensitivity to initial conditions and model accuracy. **a** Confusion Matrices of Ridge classifier in Liver Disorder Dataset on three states of Lorenz transformer: non-chaotic (stable), less sensitive to initial conditions ($\rho$ = 28), and more sensitive to initial conditions ($\rho$ = 97) **b** Color map visualization of ρ value (x-axis) LLE (y-axis) and accuracy of Linear Kernel Support Vector Machine in liver disorder dataset (color values).

In this test using the Liver Disorder dataset, we study a chaotic transformation with ρ values ranging between 1 to 100. Then, we record the best accuracy of Linear SVM. We evaluate the LLE of Lorenz attractor with ρ value in the range from 1 to 100. When compared with a non-chaotic (ρ = 2) and chaotic but less sensitive model (ρ = 28), the optimized model (ρ = 97) demonstrates higher accuracies in every single class (see Fig. 4).

We also demonstrate a positive statistical relationship between the Largest Lyapunov Exponent and model accuracy after running Welch’s t-test and Pearson’s R-value test (see Methods Statistical tests and Supplementary Note 3 for details). We report this statistical correlation with a r-value of 0.84. Such a positive correlation explains our findings in Fig. 2. The spatial clustering of samples in a dataset is enhanced by the separation pace of close points and the chaotic nature of the transformer. As points separate relatively faster, it is also more probable to find a set of clusters that we can classify linearly compared to points that do not separate. This finding is also interesting because a similar study demonstrated the necessity of chaos on classification tasks performed with neural networks^42^. In our study, we validated that chaos should be present on learning tasks whether the learning is based on conventional neural networks or reservoir computers. It should be noted that as these dynamical systems evolve, while we benefit from the separation, as mentioned earlier at early iterations, the attractor transforms the data, becoming unlearnable after a particular stage (see Supplementary Figs. 8, 9 and Supplementary Note 5).

### Circuit simulations

Encouraged by our model’s impressive performances, we study its analog implementations with circuit simulations using a specific circuit designed for the analog computation of the Lorenz attractor. After running the circuit and performing chaotic transformation to the data, we use a decision layer like our previous tests. We tune the analog computing achieved via the circuit by changing the resistance of a resistor and adjusting the ρ value. Alternatively, a digital potentiometer can be utilized to actively set the effect of chaotic data transformation in the circuit.

Our circuit simulations delivered the same performances with numerical test results, thus proving the feasibility of our proposed analog learning model (see Supplementary Table 1). In circuit simulations, we calculate the total power consumption of our analog chaotic systems. A single analog unit consumes about 350 milliwatts (to perform the chaotic transformation to data. Specifically, for the MNIST dataset, approximately 3.5 watts of power is needed for chaotic transformation. The aforementioned power consumption is two orders of magnitude smaller than conventional devices that perform machine learning (see Methods Physical model of reservoir and Supplementary Fig. 5 for details).

## Discussion

The findings of this study present a promising computing platform for the field of machine learning. The study introduces an innovative method that has demonstrated effectiveness in various machine learning applications. It notably improves power consumption for image and numerical classification tasks, using a straightforward linear last layer following a chaotic nonlinear transformation. This methodology, showcased in the context of MNIST, Liver Disorder, Iris, Abalone, and Sinus Cardinal datasets, not only enhances accuracy but also maintains input data integrity and permits flexible adjustments of model parameters and architecture. Reservoir computing studies in the literature are mostly limited to temporal predictions. Our collection of tests and step-by-step approach in machine learning tasks enables us to gradually challenge our method with tasks from temporal prediction to linear and nonlinear classification. Such tasks form a foundation and a benchmark for future machine learning models that benefit from chaos. Similar to other reservoir computing research in the literature, further specialized research may be conducted on temporal prediction in light of the results presented in this study. As presented in Sinus Cardinal regression, other configurations of chaotic transformers besides a single chaotic transformer, such as nested chaotic transformers, may be employed to enhance learning accuracy in different datasets.

One intriguing aspect of this study is the integration of circuit simulations to validate the practicality of the analog chaotic reservoir computing paradigm. This approach also enables an in-depth examination of the relationship between the Largest Lyapunov exponent of the chaotic transformer and overall model accuracy. Moreover, the circuit architecture’s speed and power efficiency on a milliwatt scale hold promise, particularly in light of contemporary concerns regarding energy consumption in machine learning applications.

Another interesting point of this study is the flexibility of our methodology. The performance of the model can increase up to 6% by altering a single parameter in the chaotic reservoir within the boundaries of the physical model. It is also an important fact that the power consumption and integrity of the physical model are independent of parameter optimization if the parameter is not set to an extreme value (ρ = 200). Although we utilize a probabilistic method of optimization to adjust our parameters in some tasks, further research may focus on different techniques of optimization to enhance learning accuracy. The Lorenz attractor, serving as the primary chaotic transformer in this study, emerges as a noteworthy element, showcasing remarkable performance in clustering and pattern recognition. The potential for further research in related areas, particularly in image segmentation using chaotic pattern recognition, is a direction that warrants exploration. The study also highlights how optimizing the chaos parameter, ρ, can lead to modest yet appreciable increases in model test accuracy. The positive correlation between model accuracy and the Largest Lyapunov Exponent raises intriguing possibilities for future research. Our method opens the door to various opportunities for further investigation, particularly in the realm of neuromorphic architectures that can harness chaos as a computational element. Similar chaotic computing techniques can be realized with silicon-on-insulator technology for chip-size footprints. Such architectures may offer innovative solutions and insights for advancing the field of machine learning.

## Methods

### Physical model of chaotic reservoir

In our method, we compute the following set of ordinary differential equations for the Lorenz attractor to transform our data^31^:

1 $$\frac{{dx}}{d\tau }= 	\, -{\sigma }x+\sigma y\\ \frac{{dy}}{d\tau }= 	\, -{xz}+\rho x-y\\ \frac{{dz}}{d\tau }= 	\, {xy}-\beta z$$

where we use coordinates x, y, and z for both input and output recording, and use parameter ρ to adjust chaos. We choose ρ because the Lorenz Attractor is more sensitive to variations in ρ, and the literature has extensively researched the attractor’s state at various ρ values. This allows us to establish our parameter search range without jeopardizing the integrity of our physical model. Due to high dimensionality, each variable is given to the circuit as an (x, y, z) vector in (variable, 1.05, -variable) format. Unless otherwise is stated in the respective section we utilize the following parameters to compute Lorenz Attractor: σ = 10, β = 8/3, and $\rho$= 97. We initially establish nodes for x, y, and z to compute the Lorenz attractor using circuits. We conduct the multiplication of nodes using analog multipliers. We initially scale the system to a large resistance to multiply nodes by their coefficients. When we intend to multiply a node by a coefficient, we add another resistor. In this manner, the value of the coefficient that multiplies the node equals the ratio between the scaling and added resistance. To iterate the system over discrete time we utilize capacitors. We are able to iterate our function across time since the voltages of capacitors are represented by a differential equation that depends on the capacitance of the capacitor. We complete the computation in a specific timestep by incorporating the resultant components via an operational amplifier within the capacitor loop. To compute the Lorenz attractor, we utilize two units of analog multiplier AD633 and three units of operational amplifier TL074. When calculated in maximum supply current and voltages, a single AD633 consumes 108 mW (±18 V, 6 A) and a single LT074 consumes 45 mW (±18 V, 2.5 A). Considering the number of units, we use; a single chaotic transformer consumes 351 mW. During all circuit simulations, we verified that supply currents, input voltages and output voltages lie within the range of the physical model and electronic components.

### Numerical simulations

For the circuit simulations, we modified the schematic of the circuit that performs the analog computation of the Lorenz system to be able to input initial conditions. We then converted this schematic to a netlist file that we will feed to LTSpice (see Supplementary Figs. 2 and 3). This netlist file consists of the circuit structure and the commands to regulate the circuit simulations. Identical to the numerical simulations, we set the timestep of the circuit simulation to 10 µs and iterated the circuit one thousand times. Afterward, we created a Python code to work simultaneously with the LTSpice simulation engine and perform parallel circuit simulations. For every variable in a sample in the dataset, this code initiates a circuit simulation after modifying the initial conditions as the variable’s value. Results of the simulations are stored in a file that will require another Python code to extract output values. This code we created retrieves one thousand iterations of every sample out of the result files and creates a matrix of output values. To complete the learning process, values are sliced iteration-by-iteration from the matrix, and the same final layers in the numerical simulations are applied to the sliced values. We retrieved power consumption data by slicing power dissipation data from the same result files.

For simulations, we created a Python code using the NumPy^43^ library that will iterate the ordinary differential equations of chaotic strange attractors in time using the Runge-Kutta method^44,45^. Identical to our circuit methodology, each variable is given to the simulation code as an (x, y, z) vector in (variable, 1.05, -variable) format. This code is then used to perform reservoir computation on the given input. Due to the high dimensionality of chaotic strange attractors, every one-dimensional predictor is transformed into a three-dimensional vector. Besides the exception of the Iris dataset, all the output vectors are used for the learning process. In the Iris dataset, after the transformation of all samples is complete, the Linear Discriminant Analysis method is applied to data before the final layer to demonstrate the decision layers and the learning process is consistent. A timestep of 10^−2^ is used to simulate strange chaotic attractors. Unless stated otherwise, the coefficients of used attractors were in their author-suggested values for the attractor benchmark test. We utilized MATLAB package of Bayesian Optimization to optimize our chaos parameters. All optimization values were used in their default state. Bayesian Optimizer minimized the prediction error. Chaos parameters are evaluated by the prediction accuracies for 100 iterations. Bayesian Optimizer scanned 20 chaos parameters to find the optimal chaos parameter.

For classification tasks (MNIST, Liver Disease, and Iris), following the same transformation process, the Ridge Classification, Linear Kernel Support Vector Machine (SVM), Polynomial Kernel SVM, Gaussian Kernel SVM, K-Nearest Neighbors, and Multilayer Perceptron Classifier algorithms are used as the last layer. All the last layers are implemented using the Scikit-learn^46^ package. Unless stated otherwise in the results or methods section, all classifiers are utilized using their default method in the scikit-learn package. The multilayer perceptron classifier utilized in the study comprises a learning rate of 10^−3^, a tangent hyperbolic (tanh) activation function, and three hundred hidden neurons.

### Data preprocessing

Excluding Sinus Cardinal data, every other dataset used was normalized using z-score normalization with a standard deviation of samples equal to one before being transformed with chaotic attractors. The Sinus Cardinal dataset is synthetically created and not normalized, with the predictor being randomly generated 2048 samples in the range of [+${{{{{\rm{\pi }}}}}}$, -${{{{{\rm{\pi }}}}}}$] and target values being the Sinus Cardinal function of generated samples. The Liver Dataset is an uneven dataset, which may result in imbalanced learning, and to prevent this, we used the Python implementation of the Synthetic Minority Oversampling Technique to upsample the Liver dataset evenly. In the MNIST dataset, we flattened the dataset and applied dimensionality reduction using Uniform Manifold Approximation and Projection for Dimension Reduction^47^ (UMAP).UMAP reduced the predictor size to 1/112 of the original data (784 to 7). Dimensionality reduction lasted about two minutes. A ratio of 80% training set to 20% test set was used to divide the datasets into training and test sets. Only for the Iris dataset, a ratio of 70% training set and 30% test set was used to divide the dataset. In all displayed results, datasets are set to training and test tests using random state zero.

For the regression tasks (Abalone and Sinus Cardinal), predictors of every sample are transformed with our code, and a simple Linear Regression algorithm is implemented as the final layer that completes the learning process. Confusion matrices are normalized over true predictions (row-wise), and decimal numbers are rounded to the nearest whole. The table results show the standard deviation of accuracies after 20 separate dataset splits.

### Statistical tests

For the Chaos and Learning test, we estimated LLEs using a well-known method in the literature^48^. We utilized the MATLAB built-in function for to estimation Lyapunov Exponents (see Supplementary Note 2 for details). We measured local LLE between iterations 1 and 100 as these iterations were our range. We decided to make parameters σ and β as fixed variates (σ = 10, β = 8/3). We decided to keep these parameters unchanged due to the high sensitivity to the initial conditions of the Lorenz Attractor, which would complicate testing. We employed the Linear SVM with MATLAB implementation for chaos and learning tests. We utilized the SciPy^49^ library functions of the given statistical significance tests.

### Supplementary information

Supplementary Materials

## Data Availability

Datasets that contain raw information are available in refs. ^37–40^.

## Code availability

All numerical and circuit simulation test data and code are available in this online repository^50^.
