# Supplementary material for: Implementing the analogous neural network using chaotic strange attractors
Source: Commun Eng. 2024 Jul 15;3:99. doi: 10.1038/s44172-024-00242-z (PMC11251050; doi:10.1038/s44172-024-00242-z)
Supplement: Supplementary file 1 — Supplementary Materials [file 44172_2024_242_MOESM1_ESM.pdf]

Supplementary Material of

Implementing the analogous neutral network using chaotic strange attractors

Bahadır Utku Kesgin and Uğur Teğin*

Department of Electrical and Electronics Engineering, Koç University, Istanbul, 34450, Turkey

*corresponding author: utegin@ku.edu.tr

Supplementary Notes 1: Creation of Input / Output Encoding

Chaotic attractors are multidimensional systems however the data we process is one-dimensional. Therefore, the creation of a stable encoding method is crucial for model stability. Initially we insert the data as the initial condition of only one dimension and kept the other two dimensions as constant variables for every predictor. We test this pipeline with Lorenz attractor, which is three-dimensional with initial conditions of (variable, 1,1). While left as a dummy variable with a value of 1, the y and z coordinates are also processed and produce unique outputs every iteration. Therefore, we start to use the output value of the dummy variables in the learning phase to benefit from the increased dimensionality and unique values of the dummy variable. In the end, our chaotic transformation method expands dimensionality from 1D to 3D for every input. After chaotic transformation with Lorenz attractor is applied to our randomly generated samples, we feed the output matrix to the regression algorithm. This process is repeated every iteration for one hundred iterations, and the best accuracy/lowest error is recorded. We record the minimum Root Mean Squared Error (RMSE) of the input method (variable, 1, 1) as 0.11. We perform series of tests with different combinations and achieved the lowest error among other tested combinations using the initial conditions (variable, 1.05, -variable) with an RMSE of 0.10.

Attractor	Sinus Cardinal Regression Minimum Root Mean Squared Error (RMSE)	Parameters
Before Transformation	0,346368	N/A
Lorenz Attractor	0,149351	a = 10 b = 2.667 c = 28
Chua's Circuit	0,187065	a = 9 b = 100/7 c = 8/7 d = 5/7
Chen's System	0,201063	a = 60 b = 2.667 c = 97
Burke-Shaw Attractor	0,234342	a = 10 b = 4.272
Sprott Attractor	0.240657	a = 2.07 b = 1.79
Rössler Attractor	0,301912	a = 0.2 b = 0.2 c = 5.7

Supplementary Table 1: Linear Regression results Sinus Cardinal regression for six selected chaotic strange attractors and before transformation

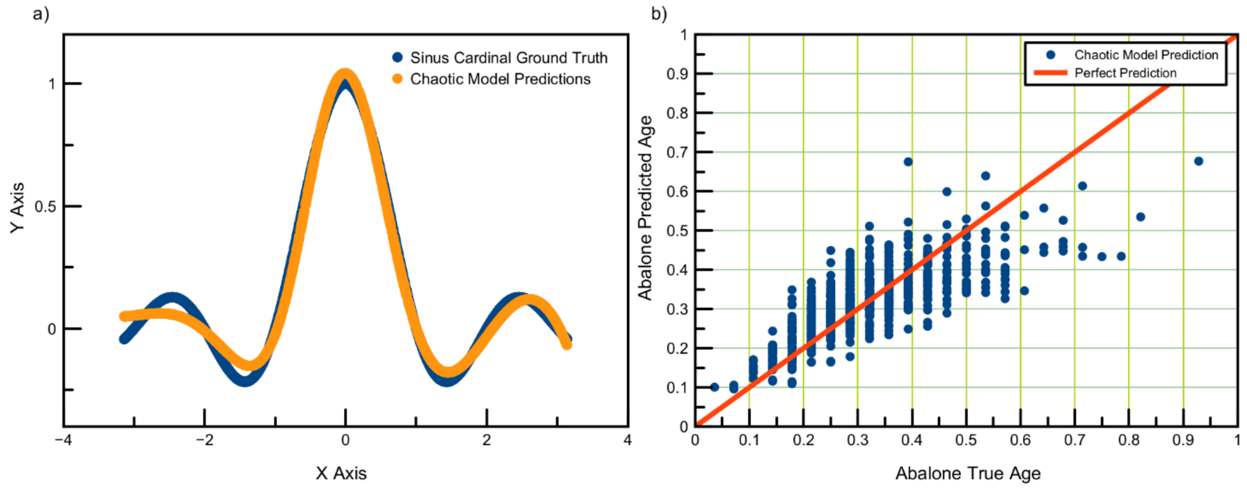

Supplementary Figure 1: Linear Regression results after the application of chaotic transformation with Lorenz attractor. a) Measured linear regression results after the application of two parallel chaotic transformations with ground truth b) Measured linear regression results of single chaotic transformation to abalone dataset.

Classifier	Liver Dataset Test Accuracies			Iris Dataset Test Accuracies			MNIST Dataset Test Accuracies		
	Before Transformation	Numerical Simulations (Iteration: 32)	Circuit Simulations (Iteration: 319)	Before Transformation	Numerical Simulations (Iteration: 98)	Circuit Simulations (Iteration: 251)	Before Transformation	Numerical Simulations (Iteration: 7)	Circuit Simulations (Iteration: 207)
Ridge Classifier	81,71%	92,82% (0,85)	92,13% (1,18)	80,00%	97,78% (2,16)	97,78% (3,47)	81,42%	95,42%	95,42%
Linear SVM	91,20%	98,84% (0,31)	97,45% (0,61)	97,78%	97,78% (3,02)	97,78% (2,35)	95,56%	95,61%	95,59%
Polynomial SVM	90,74%	88,42% (1,05)	96,30% (0,73)	95,56%	77,78% (8,74)	97,78% (3,06)	95,62%	95,58%	95,60%
Gaussian SVM	88,43%	83,79% (1,53)	93,75% (0,96)	97,78%	60,00% (7,47)	97,78% (3,09)	95,57%	95,53%	95,56%
K-Nearest Neighbors	97,69%	95,60% (0,79)	95,83% (0,61)	95,56%	91,11% (4,21)	97,78% (2,55)	95,64%	95,65%	95,65%
Neural Network	93,39%	97,22% (0,81)	98,61% (0,33)	97,78%	93,33% (3,11)	97,78% (3,78)	95,66%	95,62%	95,65%

Supplementary Table 2: Results of test accuracies of different classification algorithms in all three datasets before chaotic transformation, after numerical simulations of chaotic transformation and after circuit simulations of chaotic transformation. Chaotically transformed data is split into training and test sets for twenty times, standard deviations for every classifier are given in the parentheses. Accuracies after transformation are given in best iterations of the model.

Supplementary Notes 2: Computation of largest Lyapunov exponents

We use MATLAB's built-in function to calculate largest Lyapunov exponent. This function estimates the Largest Lyapunov Exponent using algorithm proposed by Rosenstein et.al[1]. We iterate the attractor using our iterator and apply Phase Space Reconstruction[2,3] to output data. Estimator function is applied to one-dimensional reconstructed data and estimated Lyapunov Exponents are recorded. We measure local Lyapunov exponent between our operating range. We use MATLAB for only this section due to MATLAB code being drastically faster.

Supplementary Notes 3: Statistical significance tests

We gather all data retrieved in the MATLAB code to a CSV file to interpret and feed the file to a Python code[4] that will apply Welch's t-test and Pearson's R-value test to Lyapunov Exponents and Model Accuracies. Pearson test indicated a positive correlation with a r-value of 0.84 between these two values. In Welch's t-test, we obtain a t-value of 1.35 and our null hypothesis passes with a significance level of 0.1 (80% confidence), but the fails in significance level of 0.05 (90% confidence), pointing to a mild correlation. We use Welch's t-test over Student's t-test because the means of the two data are drastically different. The degree of freedom in the t-test was two hundred.

Supplementary Notes 4: Circuit simulations

We modify the circuit schematic used to compute the Lorenz attractor in order to be able to give direct inputs. We add two voltage sources to two independent nodes (y and z) with switches to give initial input to the circuit. All constants are scaled to resistor values in 1MΩ, for example, ρ equals 1 MΩ /(R8+R9) and beta equals 1 MΩ /(R4+R5). Changing the value of R9 we modified the ρ value in our experiments. For example, when the resistance of R9 is equal to 33 kΩ, the circuit computes the Lorenz attractor with a ρ value of 28 and when the resistance of R9 is equal to 22.1kΩ, the circuit computes the Lorenz attractor with a ρ value of 42. We verify our numerical results with a ρ value of 42, this value was selected upon trying different ρ values that had Largest Lyapunov Exponents over 1.30 (highly chaotic).

In the PCB version multiplications are conducted with AD633 analog multiplier and TL074 op-amps. Our model consists of two AD633 analog multipliers and three LT074 operational amplifiers. In LTSpice simulations we didn't include these components for convenience but gave commands that are identical to the operations performed by AD633 and LT074. We verify that supply currents, input voltages and output voltages lie within the range of the physical model and electronic components.

Supplementary Notes 5: Long Iteration Tests

We applied the same learning procedure conducted in our study for 1000 iterations and recorded accuracies. As we anticipated and explained in the main text, data becomes unlearnable after attractor evolves and learning process becomes even more complex.

Supplementary Notes 6: Comparison Tests with Extreme Learning Machine

We decided to employ tests with Extreme Learning Machines to fairly compare our model with other alternatives in the literature. After same preprocessing to input data in Liver Disorder and MNIST datasets we trained ELMs in different configurations and measured respective accuracies. Then as ELM is trained based on random untrainable initial weights, we repeated this process 20 times and recorded the standard deviation of accuracies. ELMs we utilize nonlinear activation functions that facilitate learning. Although these are not used in our system, our approach surpassed ELMs in every single configuration we tested.

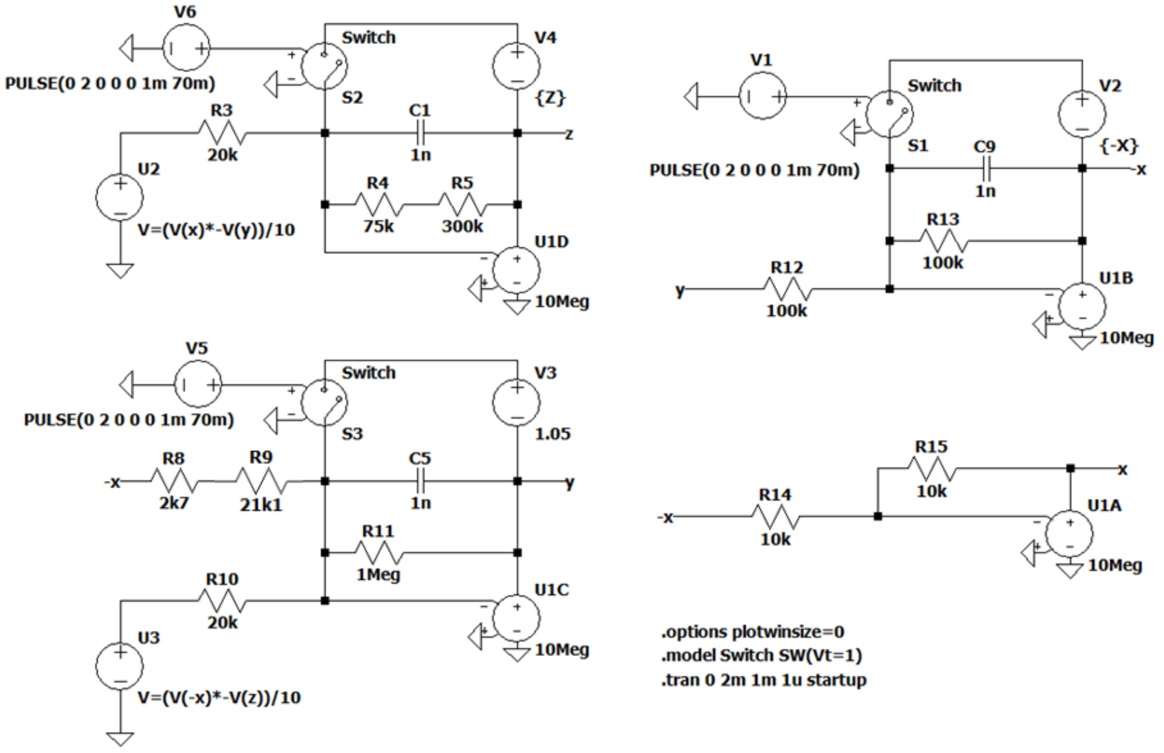

Supplementary Figure 2: Circuit schematic used in the LTSpice simulations with along simulation commands.

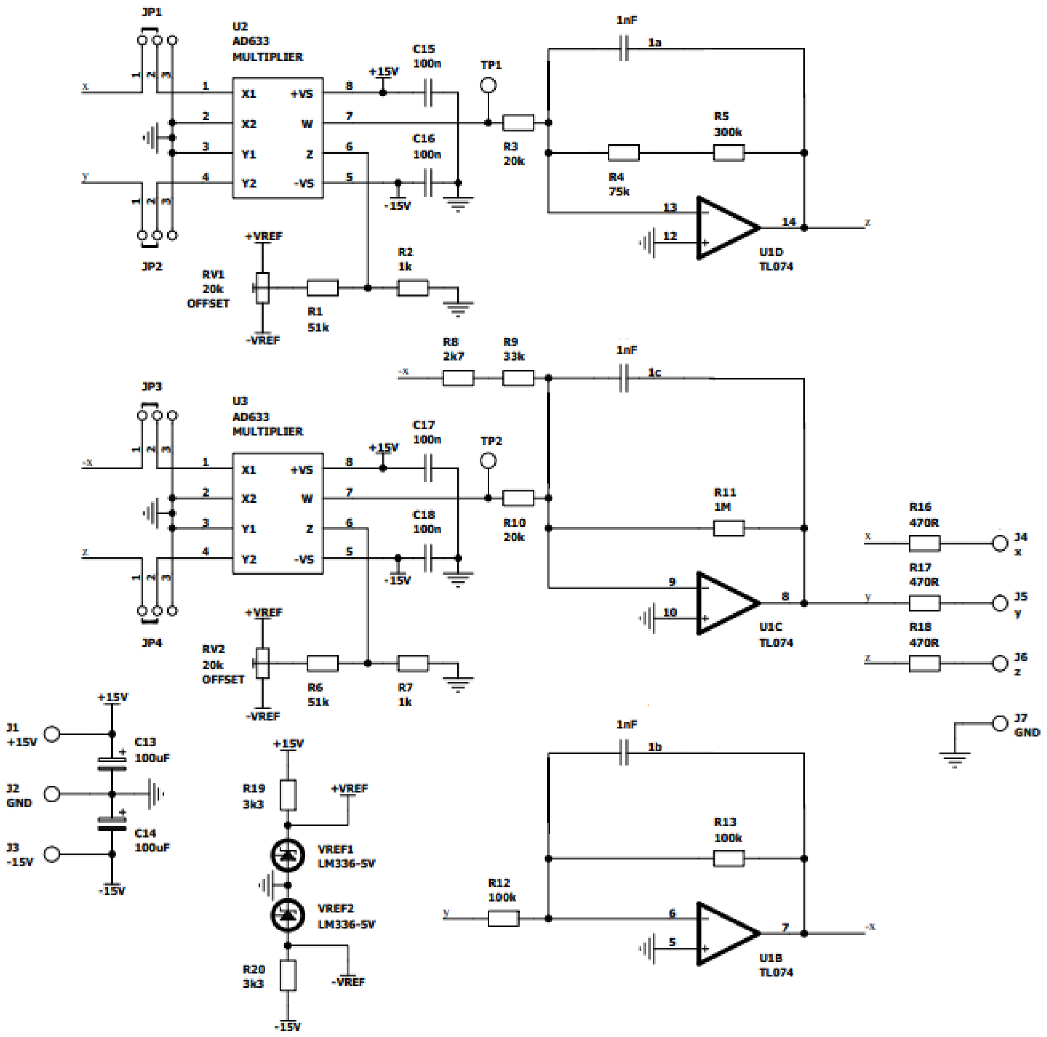

Supplementary Figure 3: Initial circuit schematic designed to use for actual analog implementation of Lorenz attractor.

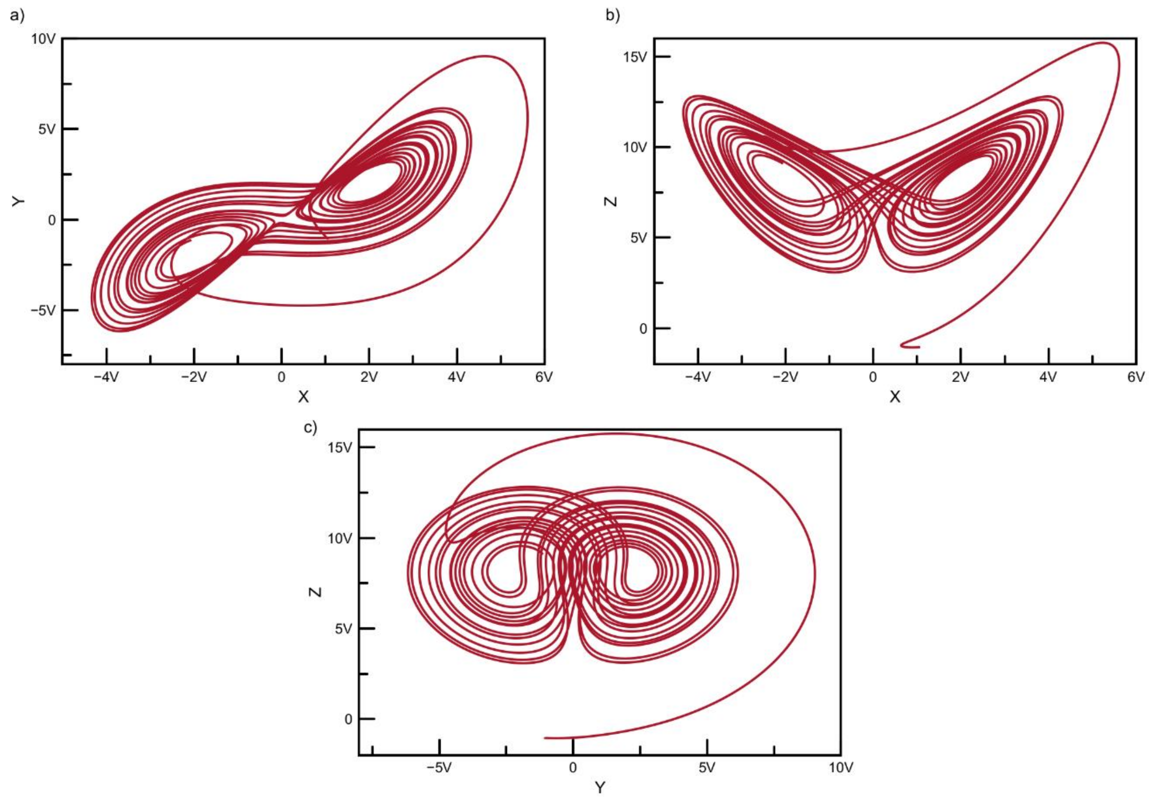

Supplementary Figure 4: Lorenz attractor computed with an analog circuit (ρ = 42), plotted in different axes: x and y (a), x and z (b), y and z (c).

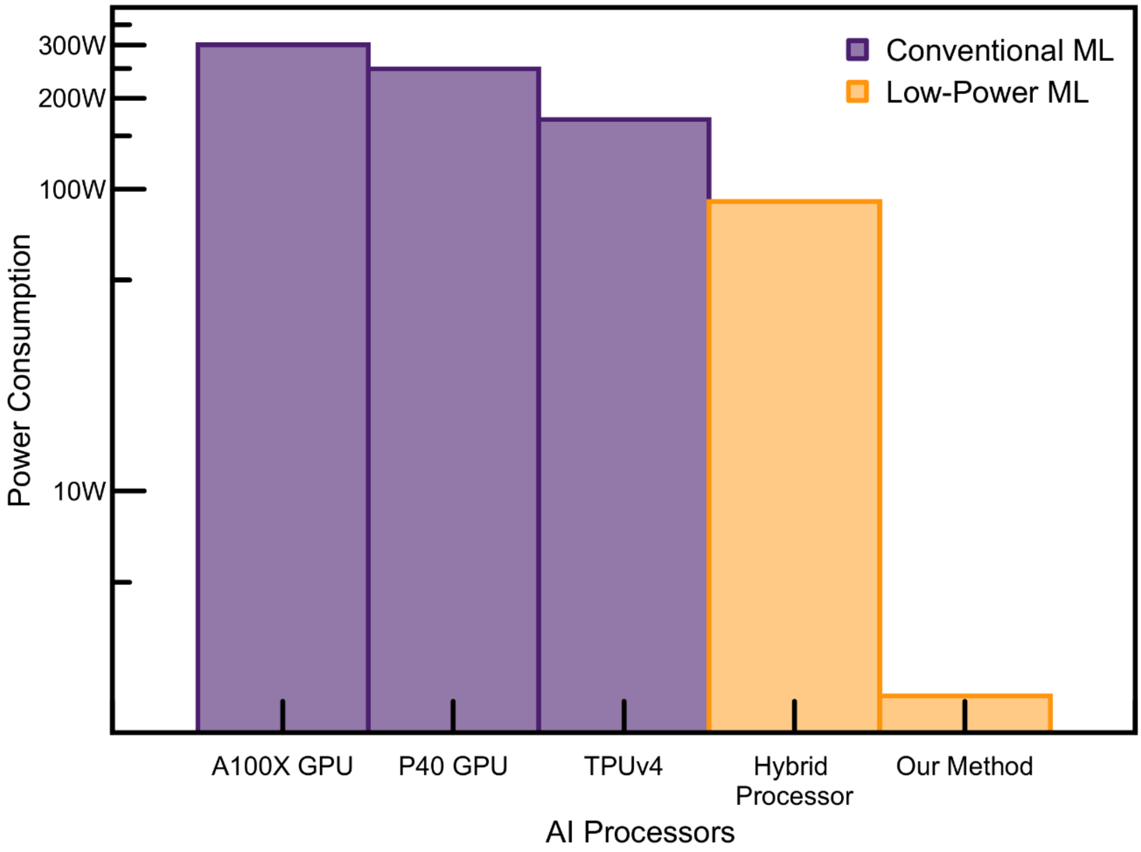

Supplementary Figure 5: Comparison of power consumption rates between conventional and low-power methods. Data of external studies taken from GPUs[5] and Hybrid processor[6]. All consumption rates are Watts per hour. Power consumption of our model is calculated based on MNIST dataset.

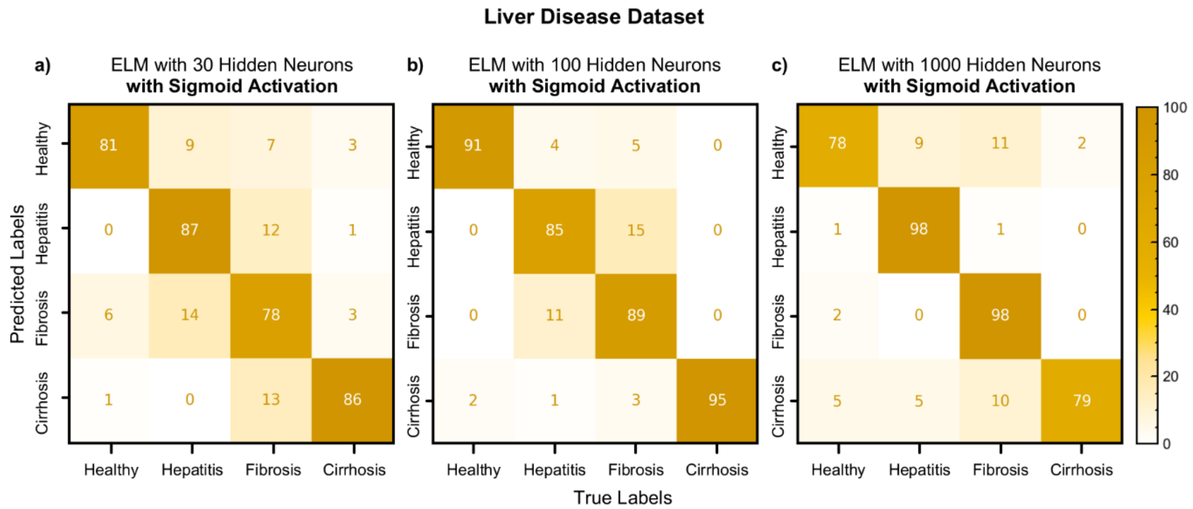

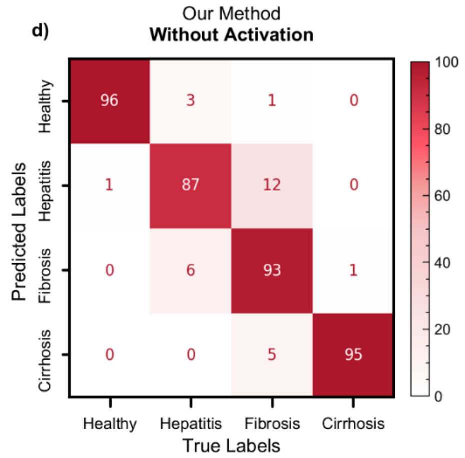

e)

Learner	Number of Trainable Parameters	Activation Function	Liver Dataset Test Accuracy
Our Method	120	None	92.82% (σ = 0.85%)
ELM - 100 Hidden Neurons	400	Sigmoid	90.04% (σ = 1.50%)
ELM - 1000 Hidden Neurons	4,000	Sigmoid	87.73% (σ = 1.32%)
ELM - 30 Hidden Neurons	120	Sigmoid	82.63% (σ = 4.75%)
All ELMs Without Activation	120<br>400<br>4,000	None	81.71% (σ = 0%)

Supplementary Figure 6: Comparison of our model with Extreme Learning Machine[7] in Liver Disease dataset. **a,b,c.** Confusion matrices based on predictions made by ELMs in different configurations. **d.** Confusion matrix of our model in its best iteration. **e.** Table illustrating classification accuracies, number of trainable parameters and used activation functions.

MNIST Dataset

Learner	Number of Trainable Parameters	Activation Function	Liver Dataset Test Accuracy (%)
Our Method	210	None	95.42%
ELM - 1000 Hidden Neurons	1,000	Sigmoid	95.00% (σ = 0.03%)
ELM - 100 Hidden Neurons	10,000	Sigmoid	94.91% (σ = 0.14%)
Memristive Neural Network	198,500	Sigmoid	88.90%
ELM - 21 Hidden Neurons	210	Sigmoid	88.51% (σ = 2.77%)
All ELMs Without Activation	210<br>1000<br>10,000	None	81.42% (σ = 0%)

Supplementary Figure 7: Comparison of our model with Extreme Learning Machine[7] and Memristive Neural Network[8] in MNIST dataset. Table illustrates classification accuracies, number of trainable parameters and used activation functions.

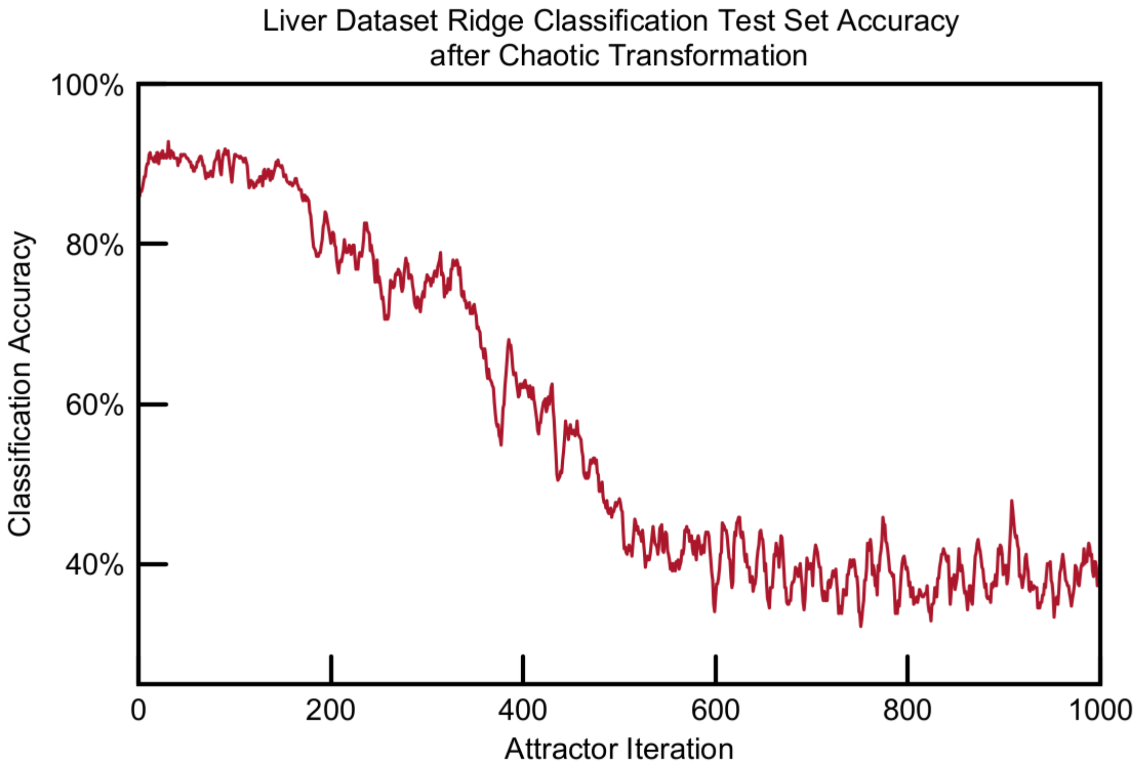

Supplementary Figure 8: Figure illustrating the test set accuracy of our model throughout 1000 iterations.

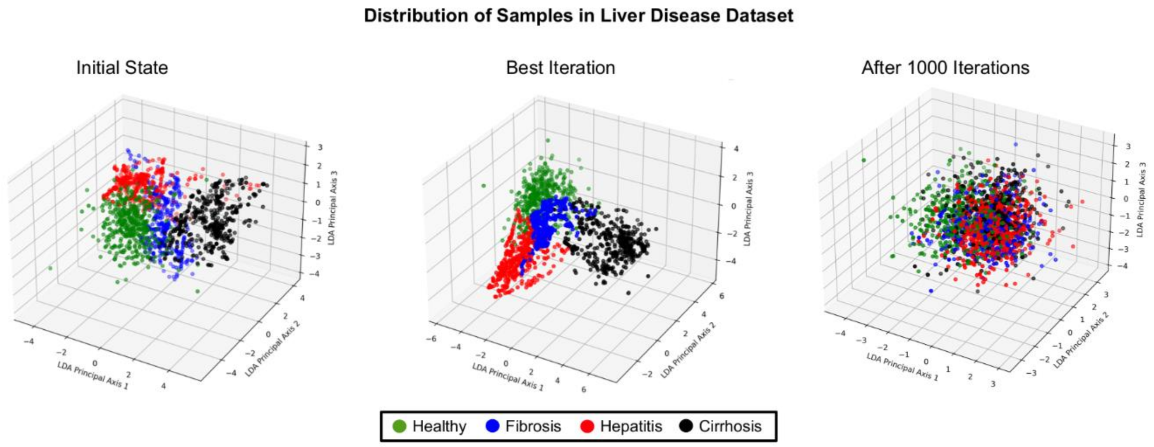

Supplementary Figure 9: Figure illustrating the sample distribution of Liver Disease dataset at the initial state, best iteration and after 1000 iterations.

Supplementary Material References

1. Rosenstein, M. T., Collins, J. J. & De Luca, C. J. A practical method for calculating largest Lyapunov exponents from small data sets. *Phys. Nonlinear Phenom.* **65**, 117–134 (1993).
2. Vlachos, I. & Kugiumtzis, D. State Space Reconstruction for Multivariate Time Series Prediction. Preprint at http://arxiv.org/abs/0809.2220 (2008).
3. Kliková, B. & Raidl, A. Reconstruction of Phase Space of Dynamical Systems Using Method of Time Delay.
4. Kesgin, B. U. bahadirkesgin/ML_w_Chaos: Final Review. Zenodo https://doi.org/10.5281/zenodo.10051449 (2023).
5. List of Nvidia graphics processing units. *Wikipedia* (2024).
6. Zhong, Z. *et al.* Lightning: A Reconfigurable Photonic-Electronic SmartNIC for Fast and Energy-Efficient Inference. in *Proceedings of the ACM SIGCOMM 2023 Conference* 452–472 (Association for Computing Machinery, New York, NY, USA, 2023). doi:10.1145/3603269.3604821.
7. Huang, G.-B., Zhu, Q.-Y. & Siew, C.-K. Extreme learning machine: Theory and applications. *Neurocomputing* **70**, 489–501 (2006).
8. Boybat, I. *et al.* Neuromorphic computing with multi-memristive synapses. *Nat. Commun.* **9**, 2514 (2018).
